# Mesenteric Lymphangioma Presenting With Small Bowel Volvulus in an Adult

**DOI:** 10.7759/cureus.16771

**Published:** 2021-07-31

**Authors:** Mohammed Barghash, Suad Nassif, Yazan Alkurdi, Moustafa Mansour

**Affiliations:** 1 General Surgery, North Manchester General Hospital, Manchester, GBR

**Keywords:** mesenteric lymphangioma, small bowel volvulus, lymphangioma, volvulus, bowel obstruction

## Abstract

Benign tumours of vascular and lymphatic origin are known as lymphangiomas. In this report, we present a case of a 26-year-old lady admitted with symptoms of small bowel obstruction. Her computed tomography (CT) scan showed a well-defined mass in the small bowel mesentery associated with small bowel volvulus. Segmental resection of the bowel, including the mass, was performed. Microscopic examination and immunohistochemistry of the specimen were consistent with lymphangioma of the small bowel mesentery.

## Introduction

Lymphangiomas are benign tumours arising from the vascular and lymphatic origin [1]. They are usually multilocular and may contain serous, serosanguinous, or chylous fluid [2]. Although lymphangiomas are frequently located in the neck, when located intraperitoneally, the small bowel mesentery has been found to be the commonest site, accounting for around 70% of cases [3].

Small bowel volvulus is one of the causes of bowel obstruction as a result of torsion of the small bowel and its mesentery. It is a well-recognised condition that might affect infants and children; however, it appears to be a rare disease in adults [4]. In this report, we present a case of mesenteric lymphangioma which presented with small bowel obstruction due to volvulus in an adult lady.

## Case presentation

A 26-year-old lady presented to the accident and emergency (A&E) department with a 24-hour history of left-sided abdominal pain. There was no associated nausea or vomiting. She also reported opening her bowels 24 hours prior to the presentation. She was a smoker (seven to 10 cigarettes per day) but denied alcohol consumption. Upon questioning, she also gave a three-month history of vaginal bleeding following a medically induced abortion.

On clinical examination, she had abdominal distension and tenderness confined to the left iliac fossa and left flank. Her blood tests showed leucocytosis; however, her liver function tests (LFTs), urea and electrolytes (U&Es), amylase, C-reactive protein (CRP), and lactate were unremarkable.

**Figure 1:**
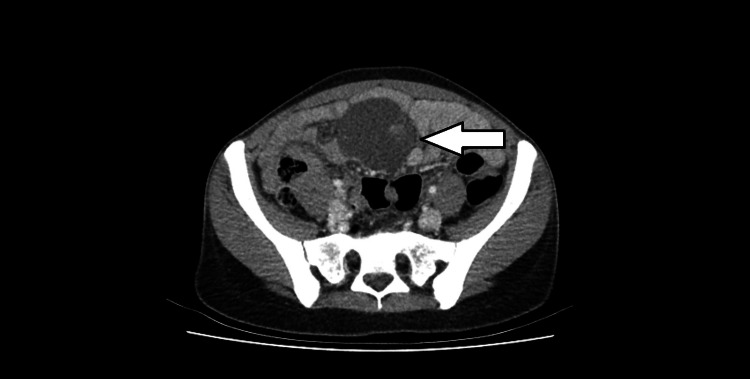
An axial abdominal CT scan image showing lobulated, fluid-attenuating mass. Mass closely abuts on small bowel loops.

**Figure 2:**
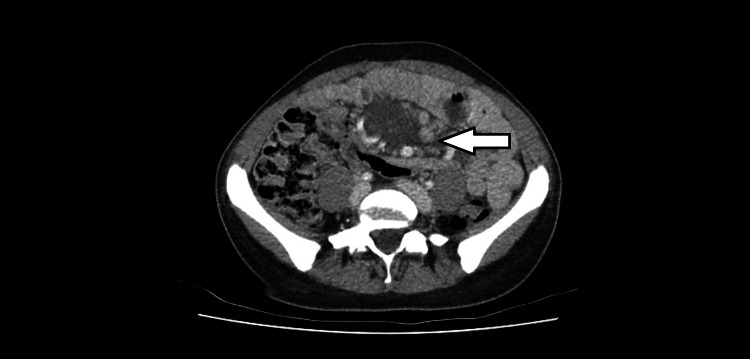
An axial abdominal CT scan image showing the mass closely related to a number of prominent vessels

**Figure 3:**
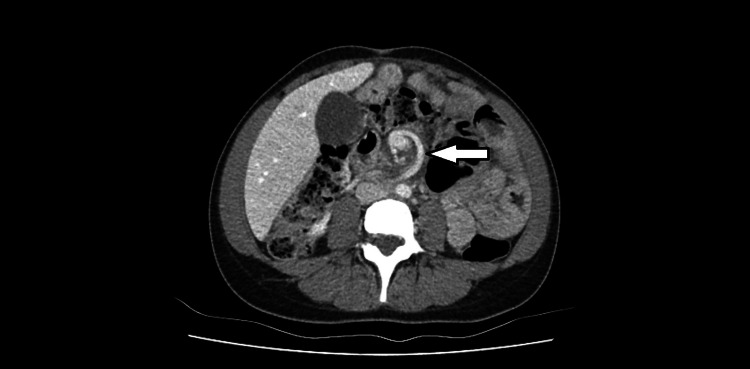
An axial CT image showing whirling of the small bowel and mesenteric vessels around superior mesenteric vein leading to its congestion

**Figure 4:**
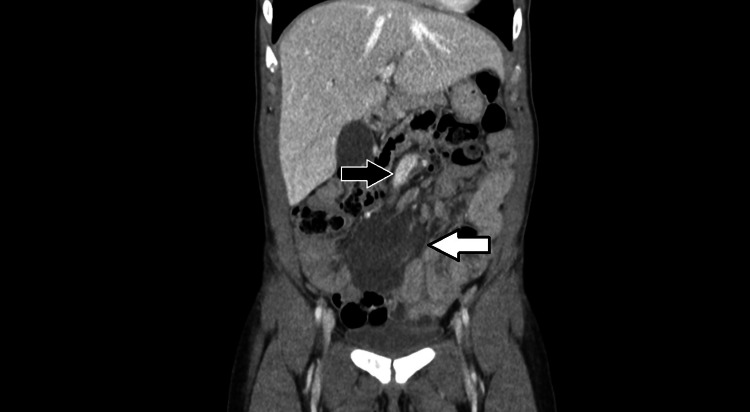
A coronal CT image showing a lobulated well-defined low-density mass with a number of prominent vessels that extend into the matrix of the lesion (white arrow). Congestion of the superior mesenteric vein (SMV) due to compression can also be observed (black arrow).

A computed tomography (CT) scan of the chest, abdomen, and pelvis was arranged and showed a lobulated well-defined low-density mass in the mesentery measuring approximately 7 cm in diameter and was associated with a number of prominent vessels that extended into the matrix of the lesion. There was also an associated small bowel volvulus with compression of the superior mesenteric vein (SMV) at the level of the mesenteric root (Figures 1-4).

After discussion and appropriate consent, the lady was taken to theatre for diagnostic laparoscopy which revealed a small amount of chylous fluid in the pelvis associated with a proximal jejunal mesenteric mass and scattered mesenteric rubbery lymph-nodes. The mass was causing a proximal mesenteric twist. Moreover, it was tethered to the root of the mesentery so laparoscopic mobilisation was unachievable. Therefore, conversion to open surgery was performed uneventfully using a midline laparotomy incision. The twist was undone and the small bowel jejunal segment was excised together with the whole mass and side to side primary anastomosis was performed using a linear stapler.

The postoperative period was uneventful. She improved steadily over a few days. She was able to tolerate oral intake and opened her bowels. Consequently, she was discharged on the fifth postoperative day with a planned follow-up in eight weeks’ time.

Microscopic examination of the specimen showed a vasoformative lesion within the small bowel mesentery, composed of dilated, thin-walled channels and cystic spaces lined by cytologically bland, flat endothelial cells and containing proteinaceous fluid along with small lymphocytes and a few red blood cells. Some of the vessel walls contained bundles of smooth muscle. Small lymphocytes were present throughout the septa separating the vessels, with the formation of numerous lymphoid follicles. The lesion expanded the mesentery, extending from just beneath the serosa up to the muscularis propria focally. The separately sampled lymph nodes showed reactive changes. On immunohistochemistry, the endothelial cells were positive for podoplanin (D2-40) and factor VII. Immunostaining for human melanoma black-45 (HMB45) and calretinin was negative. Further stains showed diffuse positivity with a cluster of differentiation-13 (CD31) within the endothelial cells, actin, desmin, calponin, and caldesmon highlight bundles of smooth muscle in the vessel walls. AE1/AE3 cytokeratins and cytokeratin 5/6 (CK5/6) were negative. A diagnosis of lymphangioma of the small bowel mesentery was concluded and a referral to the sarcoma multidisciplinary team (MDT) meeting was made. After the MDT discussion, it was agreed that there was no evidence of sarcomatous changes.

Unfortunately, because of repeated appointment cancellations by the patient, her follow-up was not possible and she was discharged back to her general practitioner (GP).

## Discussion

Cysts of the mesentery are rare. It was first described by Benevieni in 1507, yet it was not until 1884 when Rokitanski described a chylous cyst of the mesentery [5]. Although lymphangiomas are benign, they can proliferate, become aggressive, and invade into adjacent structures [6]. They are usually multilocular lesions derived from vascular and lymphatic origin and may contain serous, serosanguinous, or chylous fluid [1,2].

Approximately 95% of lymphangiomas are found in the neck and axilla and the other 5% occur in the mediastinum and abdominal cavity including the mesentery, retroperitoneum, and bones [4]. When located intraperitoneally, around 70% of cases are located within the small bowel mesentery [3]. Abdominal lymphangioma is more common in men than women, and more than 80% of cases are diagnosed during childhood [3].

The aetiology of lymphangiomas is unknown. Multiple theories have been found including embryonal origin, retention, endothelial secretory disruption, inflammatory, lymph node degeneration, trauma, surgery, and radiation therapy [3,5]. Wegner classified lymphangiomas into simple, cavernous, and cystic [3]. It is worth mentioning that only the cavernous and the cystic types have the potential for malignant transformation [3].

Histologically, lymphangiomas are differentiated from mesenteric cysts by the presence of endothelial lining, connective tissue, and smooth muscle fibres in the former and the cuboidal or columnar lining with a lack of smooth muscles in the latter [2]. Immunohistochemistry includes CD45 factor VIII-related antigen, CD31, CD43, lymphatic vessel endothelial receptor-1, vascular endothelial growth factor-3, prox-1, and monoclonal antibody D2-40, HMB-45, desmin, and calretinin [7,8].

Mesenteric lymphangioma may be discovered incidentally or manifests as intestinal obstruction, volvulus, intussusception, ischemia, mass effect, gastrointestinal bleeding resulting in anaemia, or rupture and shock [2,3,6,7,9,10]. Abdominal pain is the most reported symptom [7]. Other symptoms may include abdominal distention, nausea, vomiting, diarrhoea, constipation, or a palpable mass [2]. Differential diagnoses include mesenteric cysts, duplication cysts, ovarian cysts, hydatid cysts, tuberculosis, bowel tumours, and other intraabdominal malignancies [7,11].

The definitive diagnosis of mesenteric lymphangioma is made by histopathological examination, nevertheless, imaging may aid in the diagnosis. Sonographic examination classically shows an anechoic cyst with multiloculated and occasional echogenic debris [1]. Computed tomography (CT) of these masses demonstrates peripheral enhancement, and low or negative attenuation values correlating with the type of fluid content [3]. Magnetic resonance imaging (MRI) is the most accurate imaging modality as it allows the differentiation between mesenteric cysts and lymphangiomas [3].

Small bowel volvulus is a rare condition among adults and is usually classified into two categories [12]. The primary type occurs when there is a segmental twist of the small bowel mesentery without evidence of any predisposing anatomical abnormalities. When it is precipitated by any anatomical abnormality, it is referred to as the secondary type. These precipitating factors include postoperative adhesion, malrotation, congenital bands, intussusception, colostomy, fistula, tumors, and Meckel’s diverticulum [12]. Although rare, rotation of a large mesenteric mass can occur and lead to volvulus of the connected mesentery and small bowel resulting in a closed-loop obstruction [4].

There are two theories explaining the relation between small bowel volvulus and lymphangioma. It was argued that the flaccid and mobile nature of a mesenteric lymphangioma can result in its rotation which induces small bowel volvulus. The other theory implies that longstanding or intermittent volvulus causes lymphatic obstruction which results in lymphatic cysts. These cysts are generally unilocular cystic masses without internal septations [4].

While there have been reported cases of spontaneous regression in size of mesenteric lymphangioma, management is mainly surgical aiming at complete surgical resection whenever feasible even in asymptomatic patients, however, some advocate follow up and surveillance in incidentally discovered cases [3,6,11]. Surgery can be done by open approach as well as laparoscopically even in children and pregnant women [3,13]. In most cases, segmental bowel resection is required [3]. Some patients required pancreaticoduodenectomy as the masses were adherent to the stomach or pancreas [14]. If complete resection is achieved prognosis is excellent, yet recurrence can happen and long-term follow up with an ultrasound scan is advisable [5,11]. Sclerotherapy with multiple agents including alcohol and doxycycline had variable results ranging from complete disappearance to recurrence [3,11]. Chemotherapy and radiotherapy showed no benefit [15]. Adjuvant therapy with picibanil (OK-432) might halt additional growth in size in unresectable intraabdominal lymphangiomas [16].

## Conclusions

In this study, we presented a case of mesenteric lymphangioma associated with small bowel volvulus in an adult. Complete surgical excision along with the affected small bowel segment was achieved through laparotomy. After the histopathological and immunohistochemical assessment, the diagnosis of mesenteric lymphangioma was established. We recommend follow up with imaging such as abdominal ultrasound, CT, or MRI scans as recurrence might occur.
